# Heat stress in Africa under high intensity climate change

**DOI:** 10.1007/s00484-022-02295-1

**Published:** 2022-06-17

**Authors:** B. Parkes, J. R. Buzan, M. Huber

**Affiliations:** 1 Department of Mechanical, Aerospace and Civil Engineering, University of Manchester, Oxford Road, Manchester, M13 9PL UK; 2 Centre for Crisis Studies and Mitigation, University of Manchester, Oxford Road, Manchester, M13 9PL UK; 3 Climate and Environmental Physics (CEP), University of Bern, Hochschulstrasse 6, 3012 Bern, Switzerland; 4 Oeschger Centre for Climate Change Research, University of Bern, Hochschulstrasse 4, 3012 Bern, Switzerland; 5 Department of Earth, Atmospheric, and Planetary Sciences, Purdue University, 610 Purdue Mall, West Lafayette, IN 47907 USA

**Keywords:** Heat stress, Climate change, Evaporative coolers, Africa, Mitigation, Adaptation

## Abstract

Extreme weather events are major causes of loss of life and damage infrastructure worldwide. High temperatures cause heat stress on humans, livestock, crops and infrastructure. Heat stress exposure is projected to increase with ongoing climate change. Extremes of temperature are common in Africa and infrastructure is often incapable of providing adequate cooling. We show how easily accessible cooling technology, such as evaporative coolers, prevent heat stress in historic timescales but are unsuitable as a solution under climate change. As temperatures increase, powered cooling, such as air conditioning, is necessary to prevent overheating. This will, in turn, increase demand on already stretched infrastructure. We use high temporal resolution climate model data to estimate the demand for cooling according to two metrics, firstly the apparent temperature and secondly the discomfort index. For each grid cell we calculate the heat stress value and the amount of cooling required to turn a heat stress event into a non heat stress event. We show the increase in demand for cooling in Africa is non uniform and that equatorial countries are exposed to higher heat stress than higher latitude countries. We further show that evaporative coolers are less effective in tropical regions than in the extra tropics. Finally, we show that neither low nor high efficiency coolers are sufficient to return Africa to current levels of heat stress under climate change.

**Supplementary Information:**

The online version contains supplementary material available at 10.1007/s00484-022-02295-1.

## Introduction

Heat stress occurs when a person is unable to maintain homeostasis and subsequent healthy internal body temperatures. Extremes of heat stress cause hyperthermia and a variety of physiological factors lead to increase rate of death, such as cardiovascular disease, diabetes, and drug use (Ebi et al. 2021, and references therein). There are several methods to prevent heat stress, these include but are not limited to, reducing physical exertion in high temperatures, avoiding direct sunlight or using cooling or warming technologies to control the ambient temperature (Suzuki-Parker and Kusaka 2016). Natural responses to heat stress include sweating and dilating blood vessels in the extremities to increase radiative cooling (Simon 1993).

A person’s ability to adjust their environment to prevent heat stress is linked to their adaptive capacity. Adaptive capacity is a combination of factors and includes physiological, technological and economic options. Richer people have access to more options to adjust the environment to suit them and they have a high adaptive capacity. The inverse of this is also true, people with low adaptive capacity are by definition more vulnerable to stresses (Putnam et al. 2018; Ahmadalipour et al. 2019; Jagarnath et al. 2020). People that predominantly work outdoors are more vulnerable to heat stress as shade and cooling stations may not be available and moving indoors may not be practical. Vulnerability to heat stress is not limited to outside work, buildings may have old, inefficient or absent space cooling that prevents the occupants from altering their environment (Uejio et al. 2011). Heat stress also affects labour with extremes of temperature causing people to be less productive, or requiring commercial establishments to close to prevent injury or deaths of workers (Kjellstrom et al. 2009; Dunne et al. 2013; de Lima et al. 2021). Vulnerability is not solely determined by economic conditions, people suffering with chronic health conditions are more adversely affected by extremes of temperature (Kenny et al. 2010; Ebi et al. 2021). Climate change and an associated increase in heat stress may render some areas uninhabitable due to excessive temperatures (Sherwood and Huber 2010; Pal and Eltahir 2016).

The impacts of high temperatures on economic output were examined in Burke et al. (2015), where countries with a high average temperature are shown to experience significant reductions in gross domestic product (GDP) when temperatures rise further. Highly developed nations such as the United States of America are vulnerable to heat stress events occurring alongside power cuts. A power cut prevents the use of air conditioning which, if it is the sole cooling method, will lead to significant heat stress (Sailor et al. 2019). In less developed nations the prevalence of air conditioning is lower, which indicates reduced adaptive capacity. Concurrently, those fortunate enough to have access to air conditioning are also likely to experience power cuts. During extreme heat stress events, the excess strain on civic infrastructure can cause cascading failures where the failure of one component increases stress on another which in turn fails and increases stress on a third component. An example of a cascading failure was the 2015 Pakistan heat wave where extra demand for electricity caused the grid to fail, this greatly reduced the effectiveness of hospitals leading to further deaths (Masood et al. 2015).

The energy demand of cooling during extreme heat events is projected to increase globally into the 21^*st*^ century (Levesque et al. 2018). Furthermore, countries in the developing world, which are concentrated in the tropics, are expected to bear the brunt of this increase in energy demand (van Ruijven et al. 2019; De Cian and Wing 2019). Compound hazard events whereby a tropical cyclone is followed by a period of high temperature are projected to increase in likelihood with climate change. These events are dangerous across the globe and with the low adaptive capacity of developing countries, the effects will be felt the strongest there (Matthews et al. 2019). South Asia is projected to suffer increasing numbers of heat related deaths especially among agricultural workers who are outside in hot humid weather for much of their working day (Im et al. 2017). Cascading impacts predominantly affect regions and nations with low adaptive capacity. Inequalities in access to cooling technologies and low income are key contributors of vulnerability to heat stress (Pasquini et al. 2020; Gronlund 2014; Reid et al. 2009). Africa, with its large number of developing nations, is particularly vulnerable to heat stress and extra cooling demand (Zhao et al. 2015; Parkes et al. 2019).

The population of Africa in 2000 was 0.82 billion, and by 2050 this is projected to be 2.53 billion (United Nations Population Division 2017). Africa is also undergoing rapid urbanisation which, when combined with the population growth, leads to a significant increase in demand on civic infrastructure. Several African cities are vulnerable to heat stress under current climate conditions where temperatures can be hazardous to human health for four months of the year (Barbier et al. 2018). Urban centres are more exposed to heat stress than rural regions as a result of the urban heat island effect, which causes urban areas to be warmer than their rural surroundings (Fischer et al. 2012; Oke 1982; Tran et al. 2006; Wouters et al. 2017). As a quantitative indicator of vulnerable populations and lack of development, researchers use the Human Development Index (HDI), a combination of Life Expectancy, Education and Income (United Nations Development Programme 2010). Sub-Saharan Africa has some of the lowest HDI values on the planet (United Nations Development Programme 2019). The combination of an increasing urban population, high vulnerability, and larger temperature extremes means that Africa is particularly susceptible to future heat stress (Asefi-Najafabady et al. 2018; Ahmadalipour and Moradkhani 2018; Lelieveld et al. 2016; Sylla et al. 2018).

Climate change is projected to increase both average and extreme temperatures in Africa (Zhao et al. 2015; Sun et al. 2019; Orlowsky and Seneviratne 2012; Varela et al. 2020; Zittis et al. 2021). Higher temperatures and higher humidity are key components of increasing heat stress. The majority of cities are either coastal or near major rivers, and in the case of coastal cities, the increased humidity from evaporation further exacerbates vulnerability to heat stress (Diffenbaugh et al. 2007; Kumar et al. 2017; Coffel et al. 2017; Mishra et al. 2020). South East Asia is projected to suffer increasing numbers of days with high wet bulb temperature under future climate change (Im et al. 2018). The increase in average temperatures, alongside an increase in variability of extremes of temperature, results in people desiring more temperature control. Existing technologies such as evaporative coolers (Koca et al. 1991), colloquially known as swamp coolers, are common in the developing world. Although the target efficiency values of 65 % and 80 % are arbitrary, these represent the range that commonly used inexpensive evaporative cooling systems exhibit. Two example types of industrial scale evaporative cooling systems demonstrate the robustness of these estimates. First, the direct evaporative cooling system using a wet mesh pad that is inexpensive and has an efficiency range from 60–85 % efficiency depending on the age of the unit (Koca et al. 1991). Second, more expensive two-stage direct and indirect evaporative cooling systems, such as a mesh pad with a secondary heat pump exchanger that can be 50–110 % efficient (Heidarinejad et al. 2009). The higher efficiency system requires access to larger power and water resources. This evaporation inhibits natural sweating and also raises the local humidity, therefore the maximum cooling is limited to the wet bulb temperature (Buzan et al. 2015). Air conditioning is a forced heat transfer where rooms are cooled but the excess energy is vented outside. The electricity demand in the United States is projected to increase by 8 % under climate change (Maia-Silva et al. 2020). Similarly the cumulative financial costs of improving the electricity grid in Africa to provide energy to prevent heat stress under high intensity climate change are over $50bn by 2035 and more than $480bn by 2076 (Parkes et al. 2019).

In this study we examine the change in exposure to heat stress and the change in cooling demand in Africa using high temporal resolution output from the Coupled Model Intercomparison Project (CMIP5) climate model ensemble. We further investigate the change in cooling demand after deployment of evaporative coolers. These data sources allows us to test the following hypotheses.

1. We hypothesise that evaporative coolers will be most effective in less humid regions.
2. We further hypothesise that before the end of the 21^*st*^ century, evaporative coolers will be unable to cool regions sufficiently to replicate current conditions.
3. We hypothesise that increasing population will lead to an increase in people exposed to heat stress.

## Methods

### Climate model data

We calculate evaporative cooler algorithms and heat stress metrics using CMIP5 output (Table 1; Taylor et al. 2012). Our focus is on the Representative Concentration Pathway 8.5 (RCP8.5) for the largest climate signal (Meinshausen et al. 2011). The RCP8.5 scenario is used to develop a climate emulator that produces changes in means and extremes per degree of climate change following previously demonstrated methods (Buzan and Huber 2020; de Lima et al. 2021; Buzan 2021). Eighteen models fit the criteria with sub-daily values of temperature (*T*), moisture (*Q*), and surface pressure (*P*) across three time slices, 1986–2005 (modern), 2026–2045 (near future), and 2081–2100 (far future). The 1986–2005 span used as the modern period results from 2005 being the end of the CMIP5 historic model period (Taylor et al. 2012). 10 metre winds (*μ*_10*m*_) were only available for 14 CMIP5 models. All relevant variables are listed and described below (Table 2), and all data was interpolated to interpolated to a 0.9^∘^x1.25^∘^ resolution. The evaporative cooler algorithms are described in SI Section 1 and SI Fig. 1.

**Table 1:** CMIP5 simulations included in manuscript

Model	Reference
ACCESS1-0	Dix et al. (2013)
ACCESS1-3	Dix et al. (2013)
BCC-CSM1-1	Xin et al. (2013)
BNU-ESM	Ji et al. (2014)
CCSM4 (coupler files)	Bitz et al. (2012)
CCSM4 (lowest model level)	Bitz et al. (2012)
CNRM-CM5	Voldoire et al. (2012)
FGOALS-g2	Li et al. (2013)
GFDL CM3	Donner et al. (2011)
GFDL ESM2G	Dunne et al. (2012)
GFDL ESM2M	Dunne et al. (2012)
INM-CM4	Volodin et al. (2010)
IPSL-CM5A-LR	Dufresne et al. (2013)
MIROC5	Watanabe et al. (2010)
MIROC-ESM	Watanabe et al. (2011)
MIROC-ESM-CHEM	Watanabe et al. (2011)
MRI-CGCM3	Yukimoto et al. (2012)
NorESM1-M	Bentsen et al. (2013)

**Table 2:** Moist temperature variables and heat stress metrics

Variable or Metric	Symbol
*CMIP5 Climate Variables*	
Temperature	*T*
Surface pressure	*P*
Relative humidity	*RH*
Specific humidity	*Q*
10 m winds^1^	*μ*_10*m*_
*Moist thermodynamics*	
Wet bulb temperature	*T*_*w*_
Water vapour pressure	*e*_*RH*_
Saturated vapour pressure	*e*_*SPa*_
*Cooling Infrastructure*	
Swamp cooler efficiency 65 %	*T*_*SWMP*65_
Swamp cooler efficiency 80 %	*T*_*SWMP*80_
*Comfort and Empirical Metrics*	
Apparent Temperature	*AT*
Discomfort Index	*DI*

^1^ Only 14 CMIP5 models contain winds

### Heat stress metrics

There are multiple metrics used to estimate heat stress in humans, with some equations dating from the early 20^*th*^ century (Haldane 1905; Dufton 1929; Belding et al. 1955). A list of commonly utilised metrics along with their publication date is shown in Table 1 of Buzan et al. (2015). Two metrics were selected for use in this study, the Apparent Temperature and the Discomfort index. The Apparent Temperature (AT) is a comfort algorithm that is derived from a human thermo-physiology model (Steadman 1984; Buzan et al. 2015):

1 $$AT = T + \frac{3.3e_{RH}}{1000} - 0.7\mu_{10m} - 4$$

2 $$e_{SPa} = \frac{QP}{0.622 + 0.387Q}$$

3 $$e_{RH} = \frac{RH}{100}e_{SPa}.$$

An AT value of greater than 28^∘^C is sufficient to cause slight heat stress, a value of 32^∘^C indicates moderate heat stress with higher values increasing severity of discomfort and relevant health impacts (Zhao et al. 2015).

The Discomfort Index (DI) is an estimate of the discomfort associated with high temperatures and humidity (Thom 1959):

4 $$DI = 0.5 T_{W} + 0.5 T$$

The DI is used to estimate the cooling required to achieve a comfortable temperature with a limit of 21^∘^C, where an increase in temperature would be uncomfortable for half of the population. A DI limit of 24 ^∘^C indicates any further temperature increase causes discomfort to more than half the population (Giles et al. 1990; Sylla et al. 2018).

We calculate the fraction of the year above the AT and DI thresholds listed above. Any time the AT is above the threshold of, 28^∘^C for the lower limit or 32^∘^C for the higher limit, is a heat stress event. Similarly if the DI exceeds 21^∘^C for the lower limit or 24^∘^C for the higher limit then these are recorded as events. Solving Eqs. 1 or 4 for temperature allows calculation of the change in temperature required to bring the heat stress value below the limits (Parkes et al. 2019). This reduction in temperature is measured in degree days and referred to as cooling degree days. The use of multiple limits shows the effects of ethnic differences, genetic adaptations or acclimatisation to living and working in tropical environments (McNeill and Parsons 1999; Lee et al. 2011; Lundgren et al. 2013).

### The climate emulator, ExC-CEPt

Climate emulators are derived from a combination of statistical averaging and the use of pattern scaling techniques, machine learning, and various linear approximation statistical techniques (Lord et al. 2017; Beusch et al. 2020a, 2020b). The underlying assumption in linear regression is that the degrees of freedom are proportional to the number of data points minus one, and that the data being compared are independent. When it comes climate model output however, these assumptions are too strict because the data is dependent on underlying comparisons. Thus the degrees of freedom are much less than the total volume from the time series. Rectifying this issue, we focus on mean changes in slopes using pattern scaling (Seneviratne et al. 2012; Sillmann et al. 2013; Donat et al. 2017).

Construction of the emulator is grounded on moist thermodynamic theory (Byrne and O’gorman 2016; Buzan and Huber 2020), radiative convective quasi-equilibrium arguments (Williams et al. 2009nov; Williams and Pierrehumbert 2017), and recent developments in statistical covariance of extremes (McKinnon and Poppick 2020; Poppick and McKinnon 2020). The emulator here is in active use for a variety of applications, ranging from theoretical thermodynamic heat stress applications (Buzan and Huber 2020) and climate change induced labour reductions in agricultural settings (de Lima et al. 2021). A target variable is selected as the primary variable for which all nine other climate variables are conditioned upon. For descriptive purposes, we focus on DI. Each 20 year time series of 4x daily output for each CMIP5 simulation is ranked from lowest to highest. Following this, every other variable (Table 2) is reordered by the same index map that DI was ranked by. This provides the ranked ordered DI and conditional covariance. Due to the size of CMIP5 data ($\sim$7 TB of data), the output goes through data reduction process that extracts the statistical properties. The reordered DI time series is binned into 100 percentiles and the average and standard deviations are extracted.

Then, the baseline percentiles (1986–2005) are subtracted from the future percentiles (2081–2100). These 100 percentile differences are then divided by the global mean surface temperature change for each CMIP5 simulation, respectively. This produces the pattern scaling for which climate change projections are determined. Next, the pattern scalings between all CMIP5 simulations are averaged, producing a single DI pattern scaling map that is dependent on global mean surface temperature. Although conceptually, the RCP8.5 greenhouse gasses pathway warming is not expected to be reached at the end of 21^*st*^ Century, the amplitude of climate change is large, enhancing the pattern of warming (Buzan and Huber 2020). This allows the user to use a predetermined amount of warming such as 1, 2 or more degrees of climate change—independent of time—and evaluate outcomes (de Lima et al. 2021). This process is then repeated for the other nine conditioned variables, and produces the pattern scaling conditioned upon DI’s specific spatial pattern. This is critical, because this means that all multivariate climate projections are physically consistent. The variance between patterns within the CMIP models is small and is dependent on the unknown climate sensitivity of Earth system models (Buzan and Huber 2020; Schwingshackl et al. 2021). Finally, projections are produced by using the pattern scaling map multiplied by the amount of global mean surface temperature change plus the baseline (1986–2005) conditions. The 1986–2005 baseline has a built in + 0.6^∘^C from historical emissions (Seneviratne et al. 2016). In principle, any other time period with the same temporal and spatial resolution could be used due to the robustness and physical constraints of the patterns in Earth system models. (Buzan and Huber 2020; McKinnon and Poppick 2020; Poppick and McKinnon 2020; Schwingshackl et al. 2021).

### ExC-CEPt application

Determining the timing of air conditioning use is dependent on multiple factors: human comfort and health, evaporative cooling potential, etc. It is not enough to project, for example, the DI changes at 3^∘^C of global warming. What is required is determining if the global warming changed DI value has surpassed discomfort, labour reducing, or life threatening thresholds *after* evaporative cooling mechanisms were applied. This is where ExC-CEPt covariances are required.

Sticking with the before mentioned DI example, the entire pattern scaling of DI and the nine conditioned variables are multiplied by 3 ^∘^C global mean surface temperature change and added to the ranked DI and conditioned baselines. Then the scaled evaporative cooler output temperature, for example *T*_*SWMP*65_, is used as a new moist air temperature at 100% relative humidity. This is used to represent a new *T*_*w*_ which is combined with coincident scaled *T* to produced a new evaporatively cooled DI value. This cooled DI value is compared to the danger thresholds, and if exceeded, the 65% efficiency coolers are noted as incapable of cooling to safe values, thus requiring the more expensive high efficiency evaporative coolers. The process is repeated again with 80% efficiency coolers values, and if the cooled DI values still exceed the danger thresholds, air conditioning is required. This is applied across the 100 percentiles to produce a percentage of climatology that requires mechanical cooling at 3^∘^C global warming.

The effect of the evaporative coolers was calculated by replacing the temperature in Eqs. 1 and 4 with the adjusted temperatures. The change in temperature and evaporation of water from the coolers affects the water vapour pressure, therefore this is recalculated using Eqs. 2 and 3. Evaporative coolers saturate the atmosphere so the relative humidity is set to 100 % for use in Eq. 3 (Buzan et al. 2015). Utilising these methods warming between 0^∘^C and 5^∘^C is simulated for scenarios with no cooling, low efficiency evaporative coolers and high efficiency evaporative coolers. The control scenario is one where climate warming is 0^∘^ and no cooling technology is applied.

### Population data

The scaling approach used in this study allows analyses of large temperature changes. These temperature changes are only consistent with a high intensity Shared Socioeconomic Pathway (SSP) (O’Neill et al. 2016). Therefore the population data for this analysis are based on the total population data for SSP5 (Kriegler et al. 2017; Riahi et al. 2017). SSP5 corresponds to Fossil-fuelled Development where the world experiences high migration, strong globalisation, meat rich diets alongside no commitments to reducing fossil fuel usage (KC and Lutz 2017). The population data were regridded onto the same grid as the climate model data. We calculated average population data for four time slices, 1986–2005, 2006–2035, 2036–2065, 2066–2095, with the first using gridded historic data and the remaining three using gridded SSP5 population data. The population dependent results were calculated by multiplying the gridded population data with the gridded model outputs before summing for use in figures. This produces the total number of person-day-events. The integrated population heat stress is used to give a consistent comparison between historic and future events while incorporating both temperature and population changes.

## Results

### Apparent temperature

**Fig. 1:**
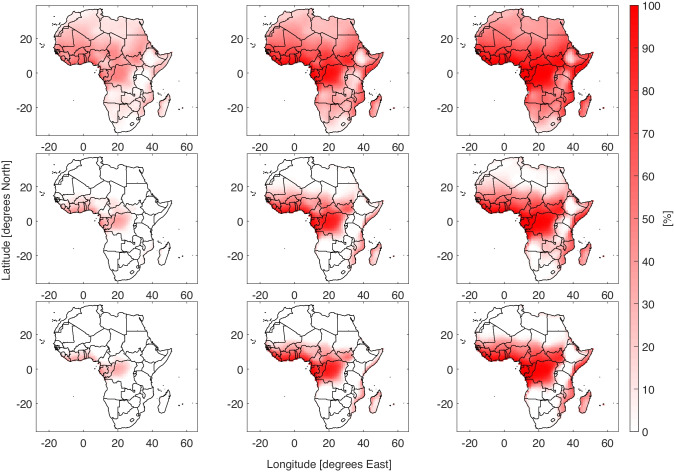
Percentage of the year where AT $\geqslant 28$ for Africa under different cooling technology and global warming conditions. The rows are from top to bottom are three cooling strategies, no cooling, low efficiency evaporative coolers and high efficiency evaporative coolers. The columns from left to right are 0, + 3^∘^C and + 5^∘^C. A version of this plot with 0 to + 5^∘^C in 1 degree stages is shown in SI Fig. 2

**Fig. 2:**
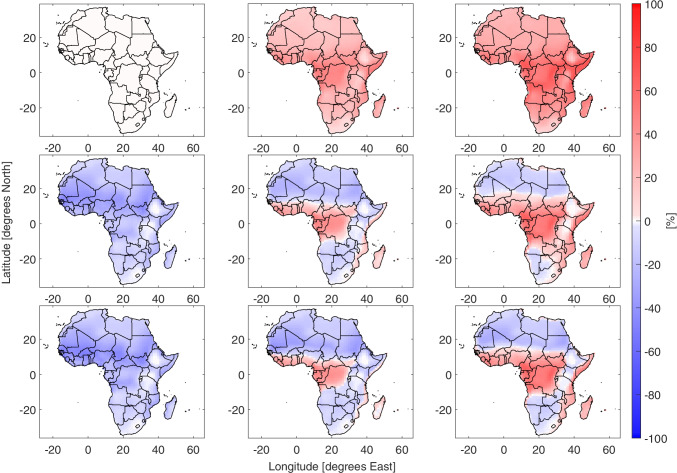
Difference in percentage of the year where AT $\geqslant 28$. The rows are from top to bottom are three cooling strategies, no cooling, low efficiency evaporative coolers and high efficiency evaporative coolers. The columns from left to right are 0, + 3^∘^C and + 5^∘^C. A version of this plot with 0 to + 5 ^∘^C in 1 degree stages is shown in SI Fig. 3

The fraction of the year where people are vulnerable to heat stress is shown in Fig. 1. There are two major patterns in the results. Firstly as climate change leads to higher global temperatures, the amount of heat stress increases, secondly there is an increase in cooling required to reduce the heat stress exposure. In the scenario with no climate change warming, the highly populated Sahel region experiences at heat stress for at least half the year. As temperatures increase the fraction of the year with heat stress (AT $\geqslant 24$) increases to the point that almost the entire year contains heat stress under high warming loads. With cooling technology of either low or high efficiency, the number of heat stress events drops quickly, especially in the extra tropics. However, evaporative coolers, which require a difference in humidity to work effectively, are of less benefit in the tropical regions of Central and West Africa. The fraction of the year where people are exposed to moderate heat stress (AT $\geqslant 32$) increases into the 21^*st*^ century (SI Fig. 8), in this case the evaporative coolers are sufficient to prevent moderate heat stress under mild warming conditions. A comparison of the number of heat stress events with the scenario without cooling technology or climate warming is shown in Fig. 2. In the Sahara Desert, there is a reduction in heat stress under cooling, this is in contrast with the increase in events in the highly populated Sahel and Central African coasts. In the case of moderate heat stress, evaporative coolers are sufficient to reduce heat stress outside of the tropics, even as temperatures reach + 5^∘^C (SI Fig. 9).

### Discomfort index

**Fig. 3:**
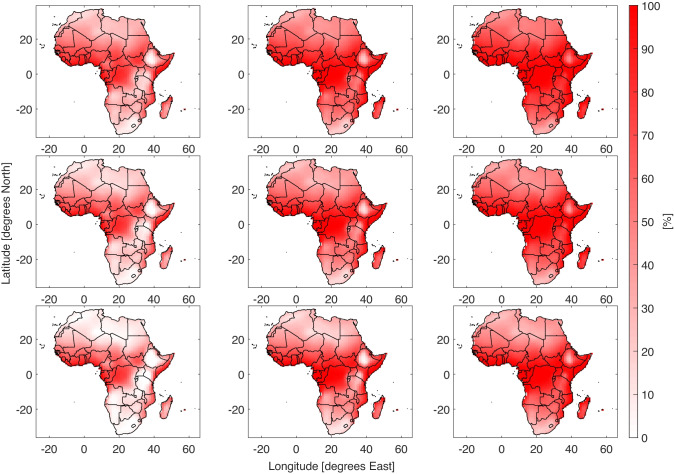
Percentage of the year where DI $\geqslant 21$. The rows are from top to bottom are three cooling strategies, no cooling, low efficiency evaporative coolers and high efficiency evaporative coolers. The columns from left to right are 0, + 3^∘^C and + 5^∘^C. A version of this plot with 0 to + 5^∘^C in 1 degree stages is shown in SI Fig. 4^1^

**Fig. 4:**
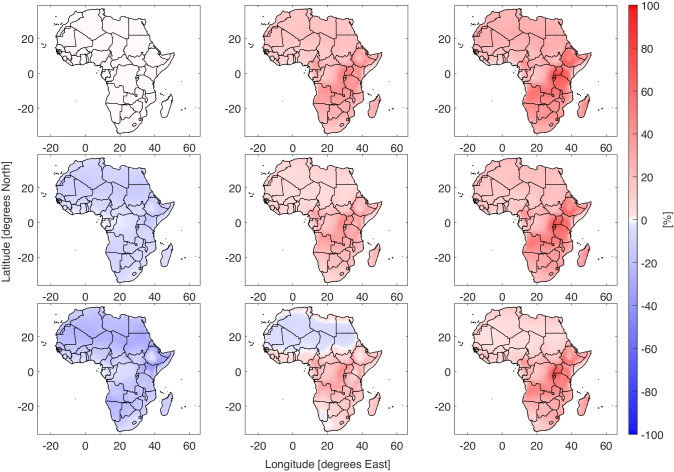
Difference in the percentage of the year where DI $\geqslant 21$. The rows are from top to bottom are three cooling strategies, no cooling, low efficiency evaporative coolers and high efficiency evaporative coolers. The columns from left to right are 0, + 3^∘^C and + 5^∘^C. A version of this plot with 0 to + 5^∘^C in 1 degree stages is shown in SI Fig. 5

The Discomfort Index and Apparent Temperature come from fundamentally different construction. Whereas the AT was the result of human physiology modelling, DI focused on comfort in relationship to cooling systems. Despite the differences in origins, similar patterns appear between the DI and AT results. The percentage of the with a DI above 21^∘^C is shown in Fig. 3. The additional discomfort events increase throughout the 21^*st*^ century and the cooling technologies reduce the amount of extra cooling required. The difference in cooling required is concentrated in the warmer equatorial regions. This pattern is repeated for the higher temperature limit results and in the historic period the high efficiency coolers almost eliminate extra cooling demand (SI Fig. 10). The difference in fraction of the year with DI above 21^∘^C from the scenario with no cooling technology nor climate warming is shown in Fig. 4. The regions where evaporative coolers are most efficient at reducing the cooling demand are in agreement with the heat stress results in Fig. 2. The dry Sahara desert requires less cooling. However this is of little impact due to the extremely low population density. Under increasing levels of climate change the cooling required increases fairly evenly across Africa. The fraction of the year with DI above 21^∘^C increases strongly in in West and Central Africa. The evaporative coolers are less effective at preventing discomfort than heat stress, particularly in the extra tropics. With an increase of 2^∘^C low efficiency coolers are overwhelmed across the continent. By 3^∘^C the high efficiency coolers cannot bring discomfort below the control scenario.

### Population dependent results

Climate change is not the only phenomena which influences the number of heat stress events, population density and population change are important for estimating the total number of events.

**Fig. 5:**
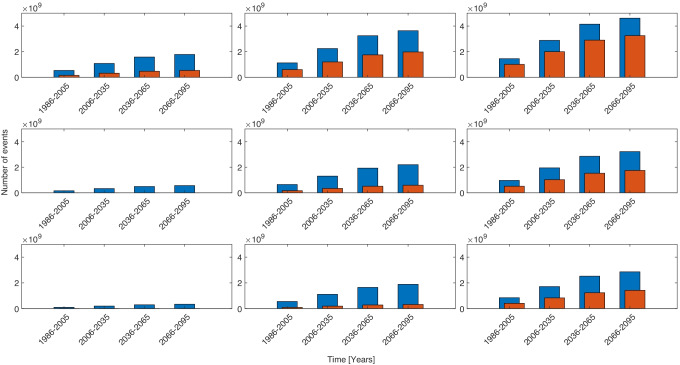
The total number of heat stress events in Africa under different cooling technology and global warming conditions. Blue bars show events with $\geqslant 28$ and orange bars show events with $\geqslant 32$. On each panel the x-axis shows the time range and the y-axis shows the number of events. The rows are from top to bottom are three cooling strategies, no cooling, low efficiency evaporative coolers and high efficiency evaporative coolers. The columns from left to right are 0, + 3^∘^C and + 5^∘^C. A version of this plot with 0 to + 5^∘^C in 1 degree stages is shown in SI Fig. 6

**Fig. 6:**
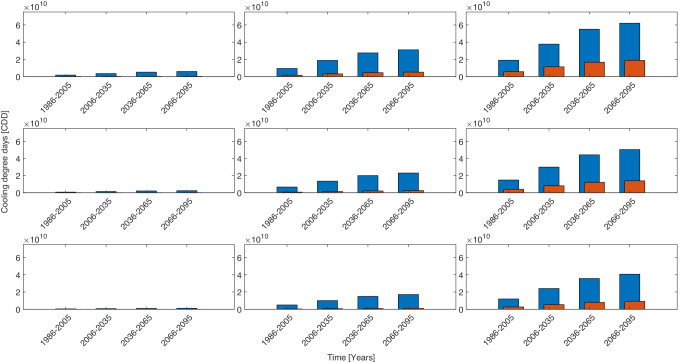
The total number of cooling degree days required to prevent heat stress in Africa under different cooling technology and global warming conditions. Blue bars show events with $\geqslant 28$ and orange bars show events with $\geqslant 32$. On each panel the x-axis shows the time range and the y-axis shows the number of events. The rows are from top to bottom are three cooling strategies, no cooling, low efficiency evaporative coolers and high efficiency evaporative coolers. The columns from left to right are 0, + 3^∘^C and + 5^∘^C. A version of this plot with 0 to + 5^∘^C in 1 degree stages is shown in SI Fig. 7

Figure 5 shows panels of the total number of heat stress events multiplied by future population change under different warming conditions and with deployment of different cooling technologies. The total number of events are heavily population driven as is shown on each bar chart, as populations increase towards the end of the century the number of people exposed to heat stress increases proportionately. The evaporative coolers are effective at preventing high levels of heat stress to the end of the 21^*st*^ century, providing global warming is kept below 2^∘^C. Figure 6 shows the number of cooling degree days, weighted by population, required to prevent heat stress. This shows that the extra cooling to prevent moderate heat stress is much smaller than the cooling required to prevent all heat stress, indicating that many events are between the two values.

### Cooling technology requirement

**Fig. 7:**
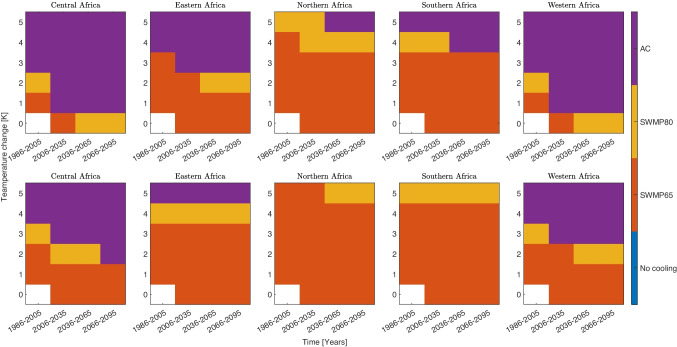
Region specific cooling technology required to reduce number of AT $\geqslant 28$ events to the amount in the control simulation for four different time periods and six potential warming levels. The control experiment is in the bottom left of each panel and is deliberately left blank. Country specific plots for AT $\geqslant 28$ events are shown in SI Figs. 12–14, AT $\geqslant 32$ events are shown in SI Figs. 15–17

The magnitude of the change in number of heat stress events on a country is a function of the population change and severity of warming from climate change. The number of heat stress events is also moderated by the prevalent cooling technology. Figure 7 shows the cooling technology required for African regions to reduce AT $\geqslant 28$ (top row) or AT $\geqslant 32$ (bottom row) events to below the number of events in the control scenario. For a country scale break down of these plots see SI Figs. 12–17. Tropical countries in West and Central Africa often have to switch to air conditioning regardless of the intensity of global temperature changes. In contrast; extra tropical nations can take advantage of evaporative coolers even with severe climate change. The high efficiency evaporative coolers have little use and many nations will be forced to move from low efficiency coolers to air conditioning to prevent heat stress.

## Discussion and Conclusions

### Robustness of CMIP5 results

The *T*_*SWMP*65_ and *T*_*SWMP*80_ values are tied to moist thermodynamics of the atmosphere, and specifically tied to *T*_*w*_. The *T*_*w*_ represents two phenomenon in the climate system. First, *T*_*w*_ is the minimum temperature that the parcel of air will cool to from maximized evaporation. Tying this to evaporative coolers, *T*_*w*_ is the equivalent to a representation of a perfect evaporator, i.e. a evaporative cooler at 100 % efficiency. Second, *T*_*w*_ is a measure of atmospheric buoyancy, and is directly tied to changes in global mean surface temperature through radiative convective quasi-equilibrium constraints (Pierrehumbert 1995; Williams et al. 2009 nov; Williams and Pierrehumbert 2017). Changes in *T*_*w*_, thus changes in *T*_*SWMP*65_ and *T*_*SWMP*80_, are shown to be robust (Buzan and Huber 2020).

### Interpretation of cooling potential

The pattern of effective cooling is linked to the moisture levels in the atmosphere, in the humid monsoonal regions of Africa, the efficacy of evaporative coolers is lower. This results in less cooling in the regions most vulnerable to heat stress. By the end of the century the regions where evaporative coolers are less effective are clearly identifiable, they are tropical West and Central Africa, along with coastal East Africa and Madagascar. Cities in the region where evaporative coolers cannot prevent an increase in heat stress include Lagos, Kinshasa, Abidjan and Dar es Salaam. These are four of the ten most populated cities in Africa. Cairo and Giza in Egypt see a reduction in heat stress when using evaporative coolers, however the impact of the Nile river may not be resolved in climate models. Recent work shows that heavy irrigated regions may exacerbate extreme heat stress (Mishra et al. 2020). Johannesburg, Nairobi and Casablanca see heat stress levels remain similar under increasing levels of climate change. This disparity of response where the richer nations of Egypt and South Africa can use cheaper cooling technology such as evaporative coolers rather than air conditioning exacerbates income inequality.

The cooling demand for comfort has a different response than the number of heat stress events. The evaporative coolers appear to prevent severe high temperatures, at the penalty of increasing humidity. This pattern breaks down by the under severe climate change of 3^∘^C and tropical Africa requires additional cooling beyond high efficiency evaporative coolers. The contrast between Figs. 2 and 4 for small temperature changes and evaporative coolers is notable as heat stress is either reduced or held stable, while cooling demand for comfort increases. It is not unreasonable to expect that people with sufficient disposable income will invest in air conditioning in the short term. If this is the case, then governments and the energy sector will need to account for the extra electricity demand during hot episodes.

The AT and DI results show similar responses when analysing both the lower and upper limits defined in Section “Heat stress metrics”. Both metrics see an expected decrease in events as the limits increase. At the continent scale for both cases, the evaporative coolers, are not sufficient to bring the number of events below those in the control scenario. However at country scale, high efficiency evaporative coolers prevent almost all moderate heat stress with the exception of the tropics, even under 5 ^∘^C of global warming.

Nigeria is an interesting case within Africa, due to its high population and tropical location Nigeria is exposed to considerable heat stress. However, Nigeria is also one of the richer countries in Africa and is therefore more able to respond to stresses on infrastructure such as increasing energy demand. The increase in infrastructure demand in Nigeria is in contrast with other affluent Africa nations such as Egypt and South Africa who will be able to continue with their existing infrastructure even as global temperatures rise. Furthermore, both nations are extra-tropical and are able to use evaporative coolers instead of more expensive air conditioning units. Countries that are both tropical and least developed are doubly vulnerable, they are exposed to significant heat stress and their existing infrastructure is often inadequate for current conditions. Countries that fall into this category include Burkina Faso, Liberia and Niger. Adding extra demand during heat stress events is dangerous and coupling this with future climate threatens the health of large portions of the population.

The urban heat island effect and its amplification of warming is too fine to be observed with the climate model analysis used herein (Fischer et al. 2012; Oke 1982; Wouters et al. 2017). Therefore the results for highly urbanised areas are likely an underestimate of total cooling demand. Increases in both temperature and urban area amplify the urban head island effect and this sprawl will increase heat stress (Marcotullio et al. 2021). The causes of increased heat stress do not solely cause heat stress. Higher temperatures increase risk of fires and droughts. Static air allows pollutants to build up and therefore reduce air quality, which will in turn negatively affect health (Heft-Neal et al. 2018; Prüss-Üstün et al. 2016). These events will not happen in isolation and compound events, where a region is struck by multiple hazards are projected to increase in frequency under future climate change (Zscheischler et al. 2018). Climate change is not restricted to temperature increase, a warmer atmosphere holds more moisture, this in turn increases thermal load on people. Focusing solely on temperature as a proxy for heat stress therefore omits part of the signal from climate change and underestimates the increase in cooling demand (Maia-Silva et al. 2020).

### Limitation of experimental design

Events where the heat stress or thermal discomfort occurs will increase in frequency with climate change. The focus of our work highlights the disparity from socio-economic access between different pathways for types of cooling infrastructure. This is accomplished by evaluating the transition from no cooling to varying infrastructure quality of evaporative coolers to the energy intensive air conditioning, which cannot be assessed through traditional methods of degree cooling days. One of the issues with using CMIP type models is that the results are grid cell averages, and do not evaluate the specific environments where humans work and live. Future work should focus on specific rural and urban environments. However, as a first estimate, evaporative coolers are not sufficient to prevent all demand for comfort cooling for low or high limits. Evaporative coolers do prevent moderate heat stress with small global temperature changes, but they are less useful in tropical regions with high humidity.

## Conclusions

Cooling infrastructure choice is a combination of health and comfortable working environment. Our analysis attacks this issue from both angels, as apparent temperature is regularly used in health applications, mitigating high heat stress is a health decision. DI is traditionally used for measuring the effectiveness of cooling to a comfortable environment, and thus mitigating high DI events is a comfort decision. The use of fans to cool an environment is a lower cost option than the evaporative coolers. They are also of limited use in the high temperature and high humidity environments in West and Central Africa. Fans moving hot, moist air have a limited window where they provide benefits to working conditions as shown in Foster et al. (2021). People with disposable income are likely to invest in their comfort, therefore even governments operating on the higher limits for AT and DI will need to provide infrastructure to support additional cooling in the near future. In the national results, the evaporative coolers are less effective in tropical regions. Tropical countries in Africa are typically poorer than their extra-tropical counterparts. The requirement of poorer nations to invest in more expensive technology and infrastructure to counteract climate change is a clear example of climate injustice (Comim 2008).

Energy systems are national or regional scale projects. Given the development time and costs, the increase in cooling demand requires active consideration by policy makers sooner rather than later. Preventing future climate change, by as little as 0.5^∘^C reduces health impacts of climate change across Africa (Sun et al. 2019).

## Supplementary Information

ESM 1 (PDF 19.9 MB)
