# Supplementary material for: Heat stress in Africa under high intensity climate change
Source: Int J Biometeorol. 2022 Jun 17;66(8):1531–45. doi: 10.1007/s00484-022-02295-1 (PMC9300535; doi:10.1007/s00484-022-02295-1)
Supplement: Supplementary file 1 — (PDF 19.9 MB) [file 484_2022_2295_MOESM1_ESM.pdf]

Noname manuscript No.
(will be inserted by the editor)

Supplementary information: Heat stress in Africa under high intensity climate change

B. Parkes · J. R. Buzan · M. Huber

Received: date / Accepted: date

1 Evaporative cooler algorithms

Calculating evaporative cooler algorithms requires a combination of T and wet bulb temperature (T_w). Our evaporative cooling algorithm follows previous methods (Buzan et al.; 2015), where we use the Davies-Jones (2008) T_w from the HumanIndexMod (a freely available Fortran routine; `https://github.com/jrbuzan/HumanIndexMod_2020`). The algorithm is an inversion of a calculation for determining efficiencies of evaporative cooling mechanisms (Koca et al.; 1991). Where instead of producing an efficiency value, we use commonly used industry standards of 65% and 80% efficiency, and back track the output temperature from the mechanism based upon CMIP5 gridcell T and T_w:

$$T_{SWMP65} = T - 0.65(T - T_w) \quad (1)$$

$$T_{SWMP80} = T - 0.8(T - T_w) \quad (2)$$

B. Parkes
Department of Mechanical, Aerospace and Civil Engineering, University of Manchester, Oxford Road, Manchester, M13 9PL, UK E-mail: ben.parkes@manchester.ac.uk

B. Parkes
Centre for Crisis Studies and Mitigation, University of Manchester, Oxford Road, Manchester, M13 9PL, UK

J. R. Buzan
Climate and Environmental Physics (CEP), University of Bern, Hochschulstrasse 6, 3012 Bern, Switzerland

J. R. Buzan
Oeschger Centre for Climate Change Research, University of Bern, Hochschulstrasse 4, 3012 Bern, Switzerland

M. Huber
Department of Earth, Atmospheric, and Planetary Sciences , Purdue University, 610 Purdue Mall, West Lafayette, IN 47907, United States

Where T_{SWMP65} and T_{SWMP80} are the output temperatures (°C) from 65% and 80% efficient evaporative coolers (respectively), T is the air temperature (°C), and T_w is the wet bulb temperature (°C). The CMIP5 gridcell T_{SWMP65} and T_{SWMP80} are an average of all environments, and cannot capture the differences between urban and rural regions, and consequently represents an estimation of environmental cooling capacity.

We show the average CMIP5 50th percentile value of T_w, T_{SWMP65}, and T_{SWMP80} over Africa for the climates with 0, +3° and +5° warming (SI Fig.1).

The CMIP5 50th percentile T_w shows dry conditions, as expected, in North and South Africa at values less than 15°C (SI Fig.1 top left). The continental interior peaks ∼21°C. At +3°C North and South Africa T_w are below 20°C, but coastal West Africa reach ∼24°C (SI Fig.1 top middle). At +5° the continental interior, and specifically coastal West Africa cannot cool below 26°C without mechanical cooling (SI Fig.1 top right).

However, modern inefficient evaporative coolers, T_{SWMP65}, show the equatorial coastal regions in West Africa only cool to ∼24°C, while in the desert regions of North and South Africa cool below 20°C (SI Fig.1 middle left). In a +3° climate T_{SWMP65} cannot cool below ∼24°C in the continental interior. Across much of the continent the T_{SWMP65} temperatures are now above 20° (SI Fig.1 centre). With temperatures increasing by 5° the inefficient evaporative coolers cannot cool the continental interiors and West and East Africa below 26°C for 50% of the year (SI Fig.1 middle right).

The modern median high efficiency evaporative coolers, T_{SWMP80}, cool below 20°C in the desert regions, but, like T_{SWMP65}, coastal regions do not cool below ∼24°C (SI Fig.1 bottom left). At +3° these coolers do are more effective than the inefficient ones and the north and south but not enough to offset the warming (bottom middle). By +5° T_{SWMP80} cools North and South Africa below 20°C, but West and Central Africa cannot cool below 26°C for 50% of the year (SI Fig.1 bottom right).

The T_w values are warm, but are not readily apparent to threaten human health or comfort. Even the T_{SWMP65} and T_{SWMP80} values in at +5°C are warm, but do not inform us if the conditions are threatening, other than 50 percent of the climatology is warmer than these conditions. The use of heat stress metrics combined with evaporative coolers results are required to determine impacts on humans.

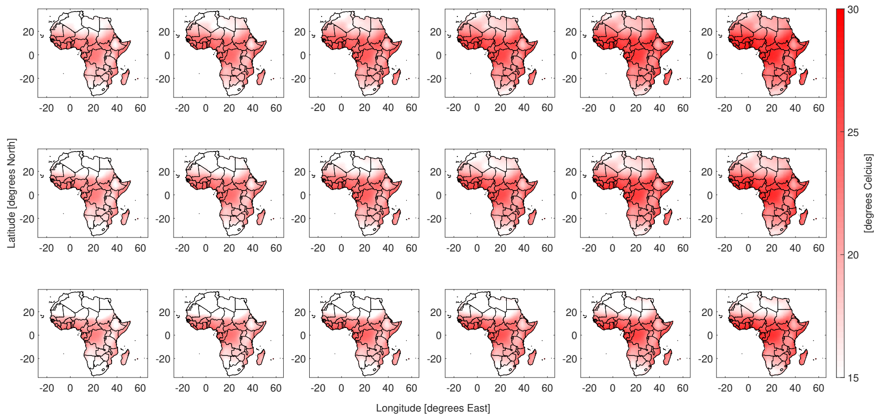

SI Fig. 1 Evaporative cooling efficiency regimes at three global mean temperature changes over Africa derived from the median CMIP5 values. Top row: low efficiency (T_{SWMP65}), middle row: high efficiency (T_{SWMP80}), and bottom row: perfect (T_w) evaporators. The columns from left to right are for 0 to +5°C in 1°C steps.

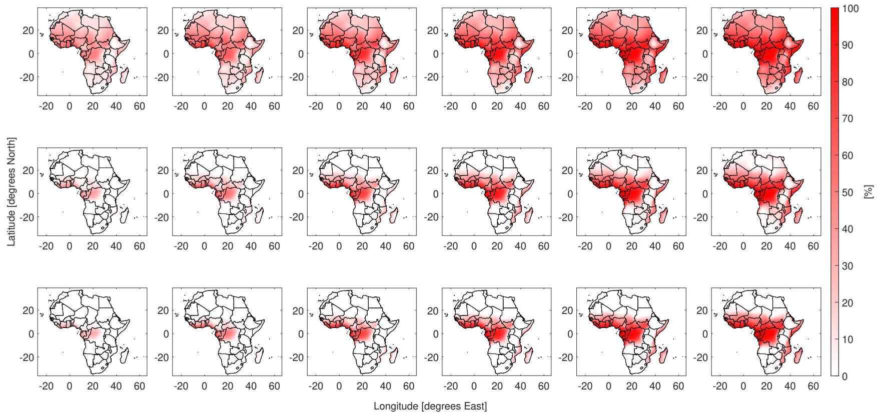

SI Fig. 2 Percentage of the year where AT $\geqslant$ 28 for Africa under different cooling technology and global warming conditions. The rows are from top to bottom are three cooling strategies, no cooling, low efficiency evaporative coolers and high efficiency evaporative coolers. The columns from left to right are 0 to +5°C in 1°C steps.

References

Buzan, J. R., Oleson, K. and Huber, M. (2015). Implementation and comparison of a suite of heat stress metrics within the community land model version 4.5, *Geoscientific Model Development* **8**(2): 151–170.

Davies-Jones, R. (2008). An Efficient and Accurate Method for Computing the Wet-Bulb Temperature along Pseudoadiabats, *Monthly Weather Review* **136**(7): 2764–2785.

Koca, R., Hughes, W. and Christianson, L. (1991). Evaporative cooling pads: test procedure and evaluation, *Applied Engineering in Agriculture* **7**(4): 485–

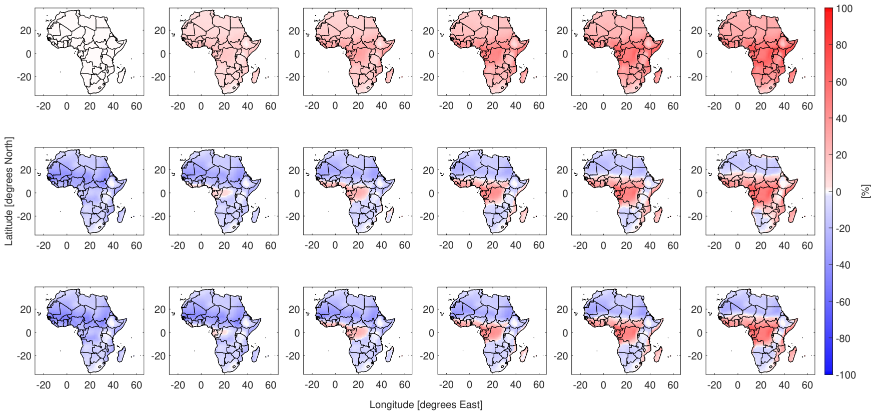

SI Fig. 3 Difference in percentage of the year where AT ⩾ 28. The rows are from top to bottom are three cooling strategies, no cooling, low efficiency evaporative coolers and high efficiency evaporative coolers. The columns from left to right are 0 to +5°C in 1°C steps.

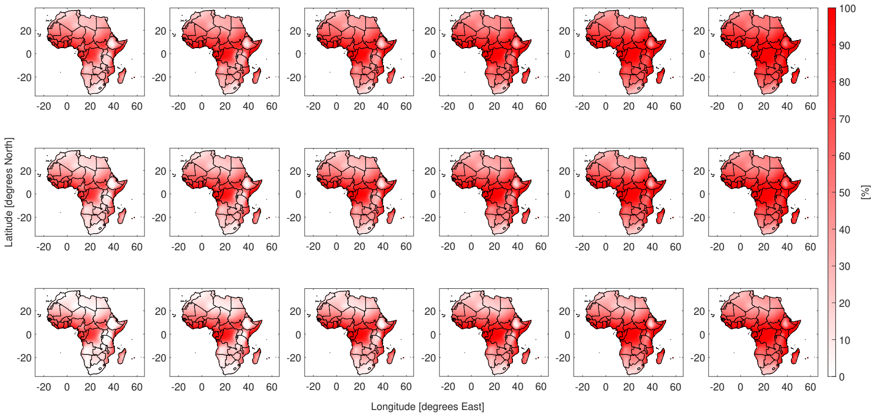

SI Fig. 4 Percentage of the year where DI ⩾ 21. The rows are from top to bottom are three cooling strategies, no cooling, low efficiency evaporative coolers and high efficiency evaporative coolers. The columns from left to right are 0 to +5°C in 1°C steps.

490.

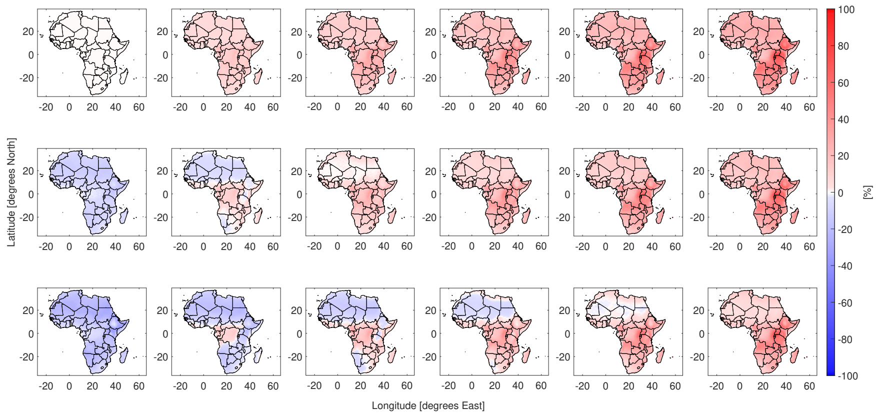

SI Fig. 5 Difference in the percentage of the year where DI ⩾ 21. The rows are from top to bottom are three cooling strategies, no cooling, low efficiency evaporative coolers and high efficiency evaporative coolers. The columns from left to right are 0 to +5°C in 1°C steps.

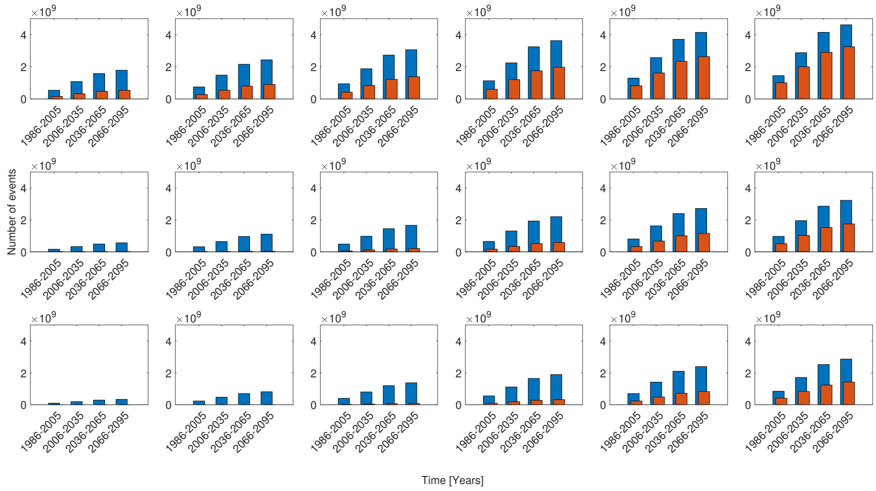

SI Fig. 6 The total number of heat stress events in Africa under different cooling technology and global warming conditions. Blue bars show events with ⩾ 28 and orange bars show events with ⩾ 32. On each panel the x-axis shows the time range and the y-axis shows the number of events. The rows are from top to bottom are three cooling strategies, no cooling, low efficiency evaporative coolers and high efficiency evaporative coolers. The columns from left to right are 0 to +5°C in 1°C steps.

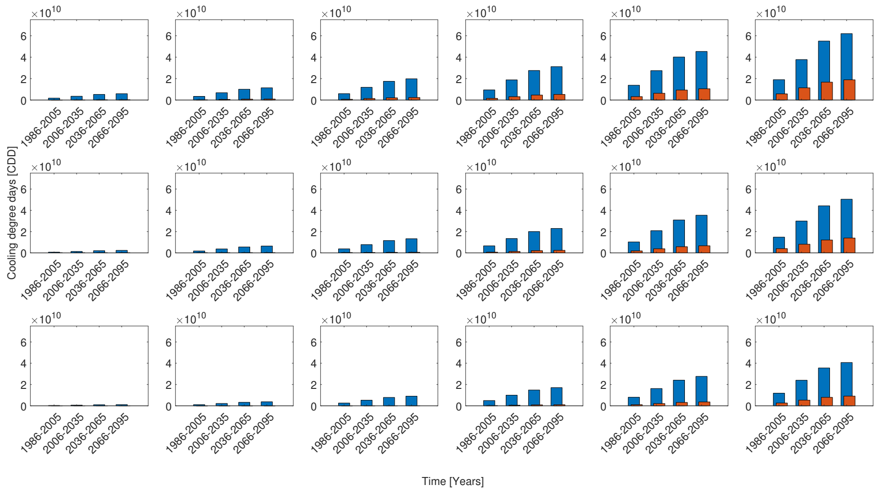

SI Fig. 7 The total number of cooling degree days required to prevent heat stress in Africa under different cooling technology and global warming conditions. Blue bars show events with $\geqslant$ 28 and orange bars show events with $\geqslant$ 32. On each panel the x-axis shows the time range and the y-axis shows the number of events. The rows are from top to bottom are three cooling strategies, no cooling, low efficiency evaporative coolers and high efficiency evaporative coolers. The columns from left to right are 0 to +5°C in 1°C steps.

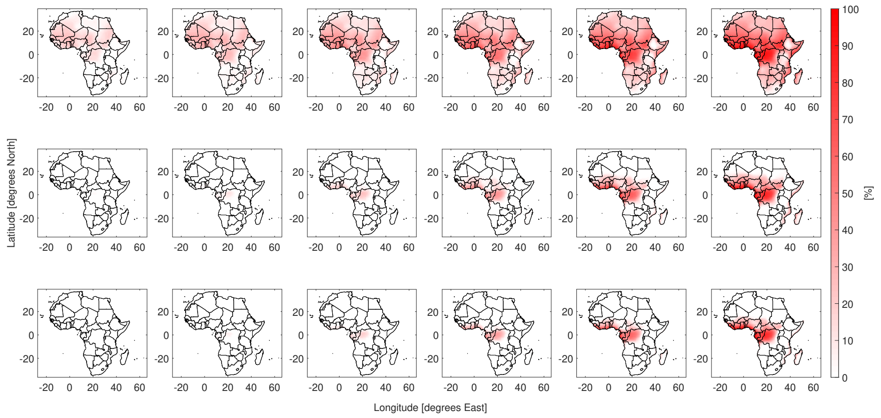

SI Fig. 8 Percentage of the year where AT $\geqslant$ 32 for Africa under different cooling technology and global warming conditions. The rows are from top to bottom are three cooling strategies, no cooling, low efficiency evaporative coolers and high efficiency evaporative coolers. The columns from left to right are 0 to +5°C in 1°C steps.

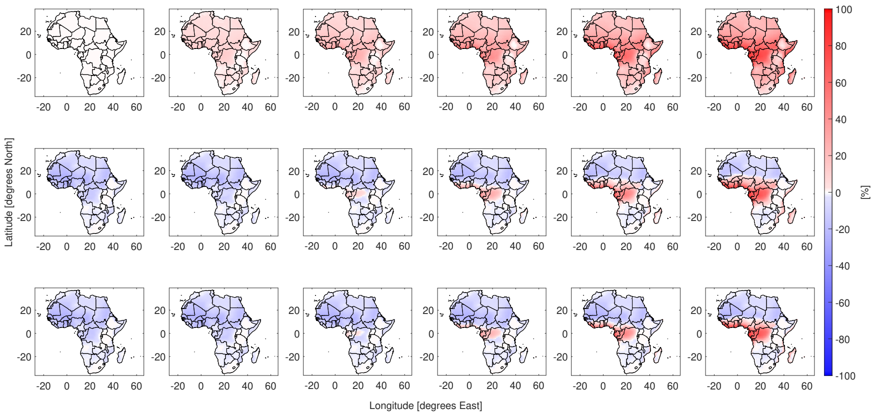

SI Fig. 9 Difference in percentage of the year where AT $\geqslant$ 32. The rows are from top to bottom are three cooling strategies, no cooling, low efficiency evaporative coolers and high efficiency evaporative coolers. The columns from left to right are 0 to +5°C in 1°C steps.

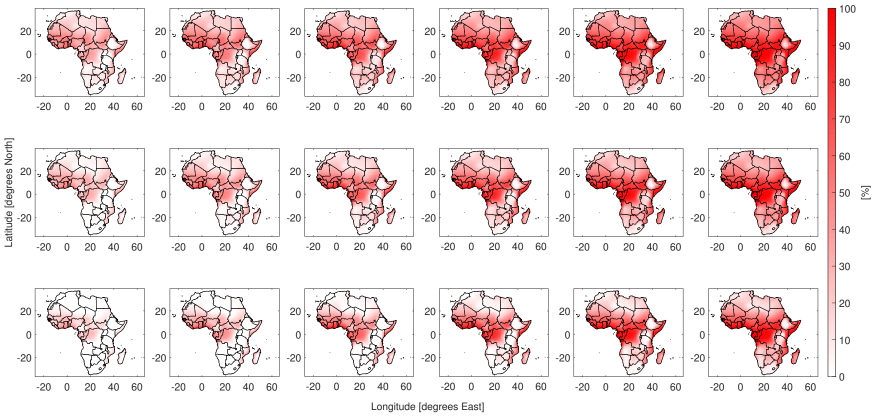

SI Fig. 10 Percentage of the year where DI $\geqslant$ 24. The rows are from top to bottom are three cooling strategies, no cooling, low efficiency evaporative coolers and high efficiency evaporative coolers. The columns from left to right are 0 to +5°C in 1°C steps.

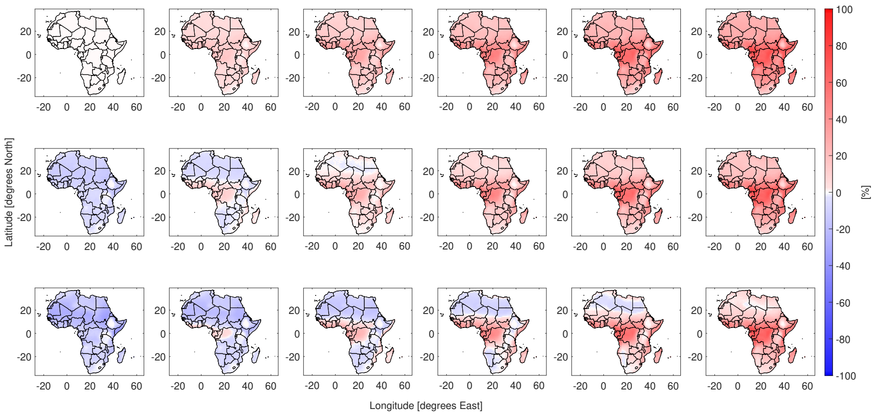

SI Fig. 11 Difference in the percentage of the year where DI $\geqslant 24$. The rows are from top to bottom are three cooling strategies, no cooling, low efficiency evaporative coolers and high efficiency evaporative coolers. The columns from left to right are 0 to +5°C in 1°C steps.

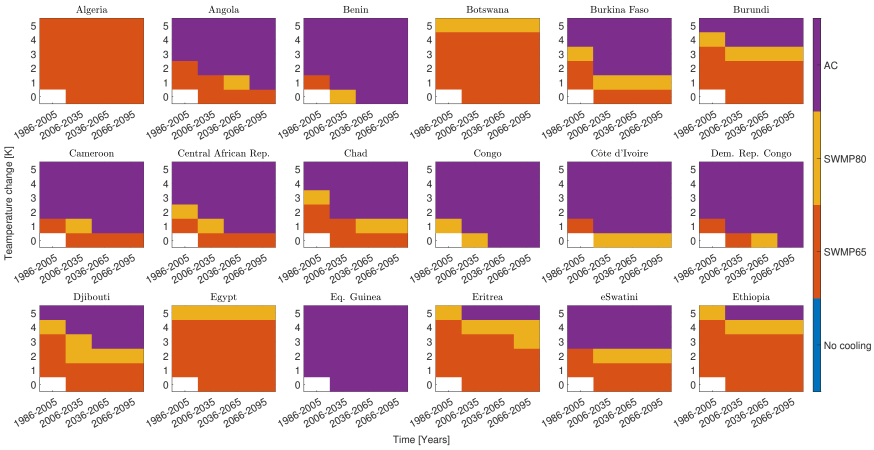

SI Fig. 12 Country specific cooling technology required to reduce number of AT $\geqslant 28$ events to the amount in the control simulation for four different time periods and six potential warming levels. The control experiment is in the bottom left and is deliberately left blank. Continued in SI Figures 13 and 14.

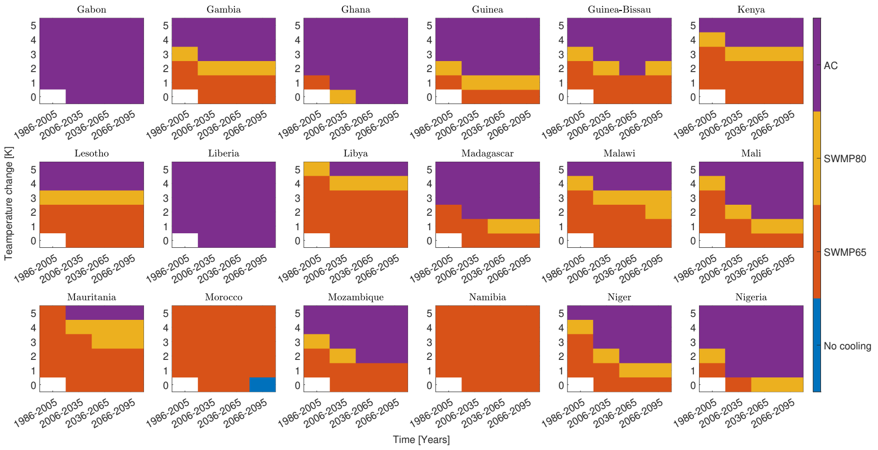

SI Fig. 13 Country specific cooling technology required to reduce number of AT ⩾ 28 events to the amount in the control simulation for four different time periods and six potential warming levels. The control experiment is in the bottom left and is deliberately left blank. Continued from SI Figures 12 and continued in 14.

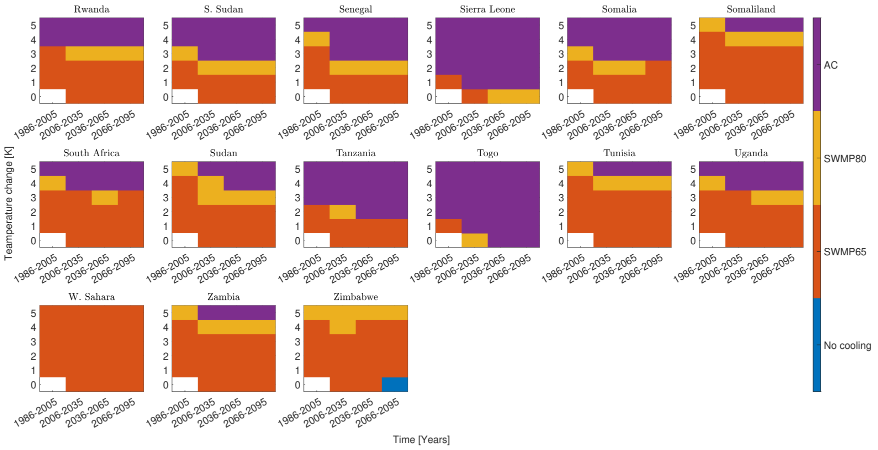

SI Fig. 14 Country specific cooling technology required to reduce number of AT ⩾ 28 events to the amount in the control simulation for four different time periods and six potential warming levels. The control experiment is in the bottom left and is deliberately left blank. Continued from SI Figures 12 and 13.

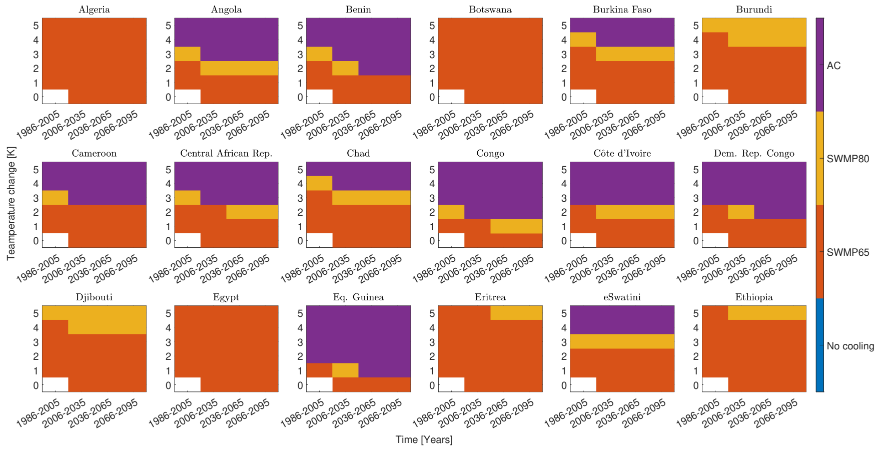

SI Fig. 15 Country specific cooling technology required to reduce number of AT $\geqslant$ 32 events to the amount in the control simulation for four different time periods and six potential warming levels. The control experiment is in the bottom left and is deliberately left blank. Continued in SI Figures 16 and 17.

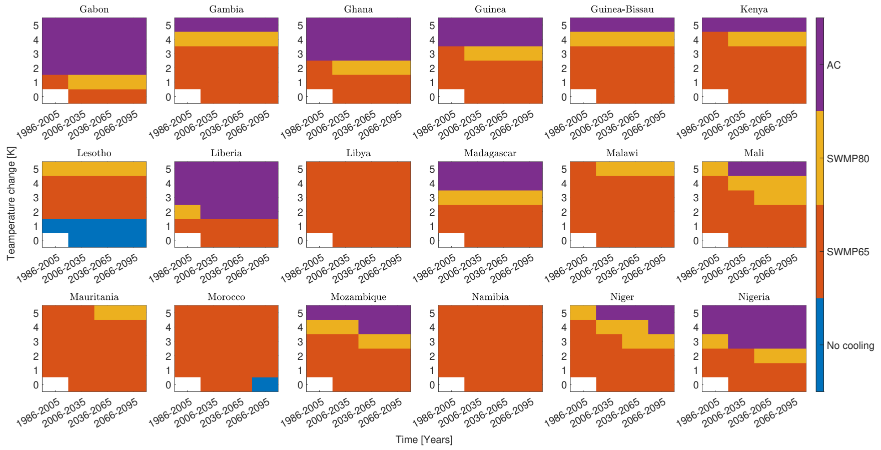

SI Fig. 16 Country specific cooling technology required to reduce number of AT $\geqslant$ 32 events to the amount in the control simulation for four different time periods and six potential warming levels. The control experiment is in the bottom left and is deliberately left blank. Continued from SI Figures 15 and continued in 17.

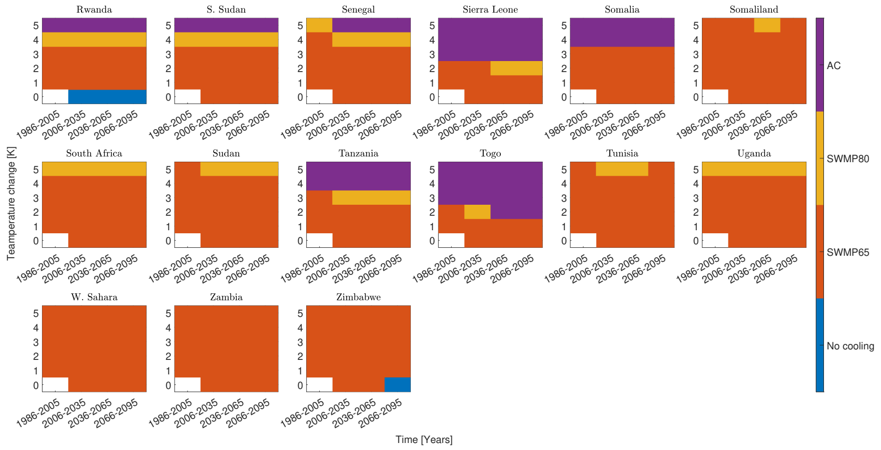

SI Fig. 17 Country specific cooling technology required to reduce number of AT $\geqslant$ 32 events to the amount in the control simulation for four different time periods and six potential warming levels. The control experiment is in the bottom left and is deliberately left blank. Continued from SI Figures 15 and 16.
